# Exploring Perceptions About Paracetamol, Tramadol, and Codeine on Twitter Using Machine Learning: Quantitative and Qualitative Observational Study

**DOI:** 10.2196/45660

**Published:** 2023-11-14

**Authors:** Federico Carabot, Carolina Donat-Vargas, Javier Santoma-Vilaclara, Miguel A Ortega, Cielo García-Montero, Oscar Fraile-Martínez, Cristina Zaragoza, Jorge Monserrat, Melchor Alvarez-Mon, Miguel Angel Alvarez-Mon

**Affiliations:** 1 Department of Medicine and Medical Specialities University of Alcalá Alcalá de Henares Spain; 2 Ramon y Cajal Institute of Sanitary Research Madrid Spain; 3 Institute of Environmental Medicine Karolinska Institutet Unit of Cardiovascular and Nutritional Epidemiology Stockholm Sweden; 4 ISGlobal Institut de Salut Global de Barcelona Campus MAR Barcelona Spain; 5 Centro de Investigación Biomédica en Red Epidemiología y Salud Pública Madrid Spain; 6 Data & AI, Filament Consultancy Group. London United Kingdom; 7 Cancer Registry and Pathology Department Hospital Universitario Príncipe de Asturias Alcalá de Henares Spain; 8 Biomedical Sciences Department University of Alcalá Pharmacology Unit Alcala de Henares Spain; 9 Immune System Diseases-Rheumatology and Internal Medicine Service Centro de Investigación Biomédica en Red Enfermedades Hepáticas y Digestivas University Hospital Príncipe de Asturias Alcala de Henares Spain; 10 Department of Psychiatry and Mental Health Hospital Universitario Infanta Leonor Madrid Spain

**Keywords:** awareness, codeine, machine learning, pain, painkiller, perception, recreational use, social media, twitter

## Abstract

**Background:**

Paracetamol, codeine, and tramadol are commonly used to manage mild pain, and their availability without prescription or medical consultation raises concerns about potential opioid addiction.

**Objective:**

This study aims to explore the perceptions and experiences of Twitter users concerning these drugs.

**Methods:**

We analyzed the tweets in English or Spanish mentioning paracetamol, tramadol, or codeine posted between January 2019 and December 2020. Out of 152,056 tweets collected, 49,462 were excluded. The content was categorized using a codebook, distinguishing user types (patients, health care professionals, and institutions), and classifying medical content based on efficacy and adverse effects. Scientific accuracy and nonmedical content themes (commercial, economic, solidarity, and trivialization) were also assessed. A total of 1000 tweets for each drug were manually classified to train, test, and validate machine learning classifiers.

**Results:**

Of classifiable tweets, 42,840 mentioned paracetamol and 42,131 mentioned weak opioids (tramadol or codeine). Patients accounted for 73.10% (60,771/83,129) of the tweets, while health care professionals and institutions received the highest like-tweet and tweet-retweet ratios. Medical content distribution significantly differed for each drug (*P*<.001). Nonmedical content dominated opioid tweets (23,871/32,307, 73.9%), while paracetamol tweets had a higher prevalence of medical content (33,943/50,822, 66.8%). Among medical content tweets, 80.8% (41,080/50,822) mentioned drug efficacy, with only 6.9% (3501/50,822) describing good or sufficient efficacy. Nonmedical content distribution also varied significantly among the different drugs (*P*<.001).

**Conclusions:**

Patients seeking relief from pain are highly interested in the effectiveness of drugs rather than potential side effects. Alarming trends include a significant number of tweets trivializing drug use and recreational purposes, along with a lack of awareness regarding side effects. Monitoring conversations related to analgesics on social media is essential due to common illegal web-based sales and purchases without prescriptions.

## Introduction

More than 25 million adults are affected by chronic pain in the United States [1,2]. The prevalence rate of chronic pain varies between 11% and 40% [3]. The treatment for chronic pain is complex, and the majority of guidelines recommend a psychological approach as well as pharmacological treatment [4-6]. The pharmacological treatment includes nonopioid as well as opioid medication [3].

Over-the-counter (OTC) drugs are generally effective and safe; thus, they are used for the control of mild pain when no medical consultation is needed [7]. Paracetamol is widely used for the treatment of mild pain, with a good safety profile when used as recommended; however, there are increasing reports of it being used inadequately [7,8]. In a study analyzing OTC drugs, which included paracetamol, 24% of adults confirmed that they took a higher dose than the recommended [9]. Another study found that many people do not pay attention to the information in the pharmacological leaflet, and more than 50% are unaware of the active ingredient they are taking [10]. Codeine and tramadol form part of the weak opioid group; they are not exempt from secondary effects, and on many occasions, they can be obtained as OTC drugs [11].

Moreover, despite being illegal, the sale of prescription medication on the internet has been detected [11,12]. Twitter represents a platform where criminal agents can commercialize and sell these drugs. Sales through Twitter are subject to lower standards of regulation and supervision than other web-based platforms [13].

The investigation of beliefs and attitudes of patients has traditionally been investigated with surveys, interviews, and clinical trials [14,15]. Recently, investigators have been making more use of social media platforms for the monitoring of public health, the study of attitudes toward treatments, and to increase the knowledge of the medical experience of patients [16-18]. Twitter is the most used social media platform for health research for many reasons, namely due to the public feed users have.

Twitter has been effectively used in analyzing social stigma toward specific circumstances such as obesity or mental health [19,20], as well as in the field of health education [21], including dental health, tobacco and vaping [22,23], and vaccinations [24,25]. There is also evidence of a correlation between Twitter posts and real-life clinical events, such as suicides or substance and alcohol abuse [26-28]. In this context, the exploration of tweets discussing the perception of drugs for better comprehension, complementation, and therapeutic decision-making has been thoroughly investigated in many areas of medicine, including opioid medication [29-31].

The main objectives of this study are (1) to conduct a quantitative analysis of Twitter posts from the years 2019 and 2020 regarding weak opioids and paracetamol; (2) to characterize the user profile that engages the most in these conversations; (3) to identify the most frequently discussed medical and nonmedical topics; and (4) to pinpoint the topics and user types that generate the most interest. Our hypothesis is that paracetamol will be the most tweeted drug due to its widespread use among the general population, that health care professionals will have the highest engagement in these discussions, and that the most discussed medical aspect will be the drug’s efficacy, while the most discussed nonmedical aspect will be its price. Last, we hypothesize that posts published by health care professionals will generate the most interest among Twitter users.

## Methods

### Data Collection

In this quantitative and qualitative observational study, we have focused on tweets that referenced the weak opioids tramadol and codeine. Paracetamol was chosen as the control drug due to its efficacy in different types of pain, its safety profile, its extensive clinical use in a wide range of patients, and its availability and accessibility. For that, we have collected all the tweets making reference either to the active ingredient or commercial names approved in Spain or the United States of paracetamol, codeine, or tramadol: paracetamol, acetaminophen, tramadol, adolonta, capdol, captor ceparidin, diliban, dolodol, enanplus, paxiflas, pazital, tioner, tracimol, tradonal, conzip, rybix, odt, ryzolt, ultram, codeina, codeine, codeisan, histaverin, notusin, and perduretas y tuzistra xr. The tweets had to meet the following requirements in order to be included: (1) posted from an open account, (2) contain a keyword from the list mentioned above, (3) have been published between January 2019 and December 2020, and (4) be written in either Spanish or English. We also collected data complementary to the tweets: the number of retweets and likes generated by each tweet, as well as the description of the user’s profile. The tool used for the collection of tweets was Tweet Binder, which allows access to 100% of public tweets.

### Process of Content Analysis

**Figure 1:**
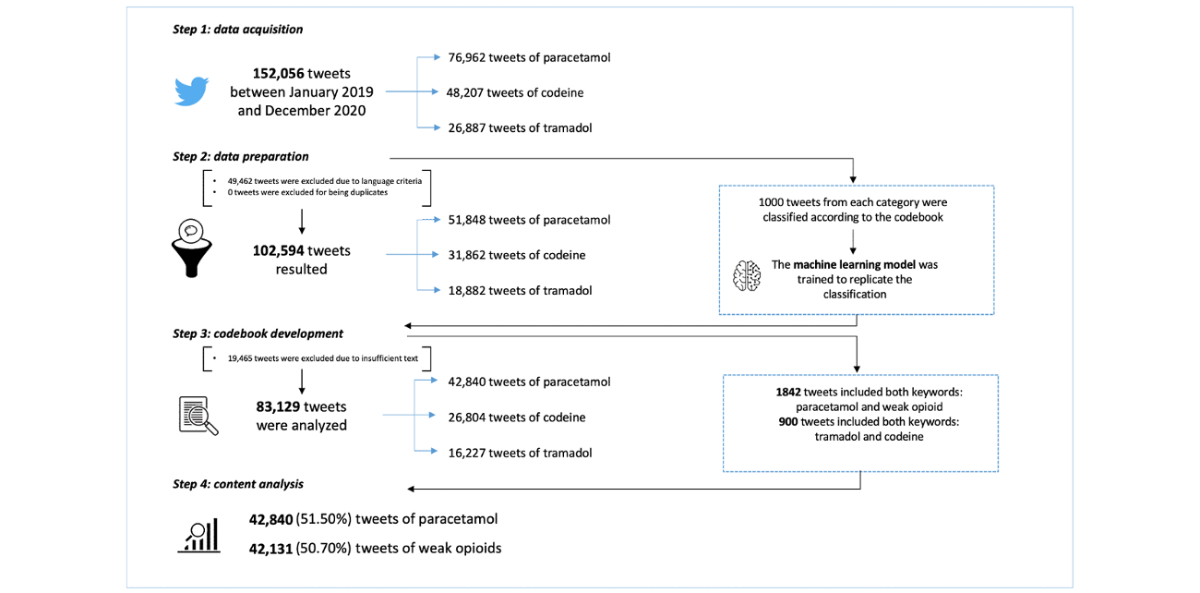
Flowchart of the study design.

A total of 152,056 tweets were collected; of those, 49,462 were discarded due to the tweet being written in a language different from English or Spanish (Figure 1). The investigators created a codebook to analyze the tweets. Modifications in the codebook were carried out after an analysis of 300 tweets by 3 investigators to elaborate the final codebook. The interrater reliability between raters was assessed, obtaining κ values ranging from 0.71 to 0.89 for the different categories. In the codebook, the type of user is the first domain classified, and then it distinguishes between medical and nonmedical content. We determined that a tweet should be classified as “medical content” when it made a clear allusion to the efficacy, adverse effect, or dissemination of the drug. Also, we have classified the tweets that included personal opinions, distinguishing those that had a positive message from those that had a negative one. Finally, we identified those tweets that pose questions. In terms of the type of user, we distinguished between patients, family members and friends, health care professionals, or institutions. To determine the type of user, we examined the Twitter profile of the publisher (useful when identifying health care professionals or institutions), the pronouns used (useful to distinguish patients and family members or friends), or the content of the tweet itself (for example, it is common for patients and health care professionals to reveal themselves as such). In terms of the content, if it was of a medical nature, we classified it depending on whether it made reference to the efficacy of a drug or its adverse effects. We also analyzed if the information stated was scientifically correct, identifying those tweets that included links to scientific papers. Moreover, in the nonmedical content, we distinguished between four themes: (1) commercial issues, (2) economic aspects, (3) solidarity, and (4) trivialization. Finally, looking at the active ingredient, we classified the tweets into 3 categories: paracetamol, tramadol, and codeine. We selected a total of 1000 tweets for each drug, which were classified manually.

### Multilingual Machine Learning Classifier

The goal of this initial tagging of 1000 tweets was to provide the data to train, test, and validate machine learning classifiers so that all the extracted tweet classifications could be inferred. To train the classifiers, a transformer multilingual model, xlm-roberta neural net, was used, and the library to deploy it was *ktrain* [32,33]. The following additional features were generated to improve the understanding of the selected set: the number of tokens that the sentence contains, the total length of the tweet in characters, the language of the tweet, and the extracted hashtags from the tweet. Also, to improve the machine learning classifier performance, we generated a clean text that takes the mentions (@) and hyperlinks out of the tweet so that it is more readable and less noisy.

Out of the 1000 manually labeled tweets, we reserved 10% (100/1000) to use as a blind set for model validation, and then the setup for training the classifier was 80% (800/1000) training and 20% (200/1000) validation. First, the training was done on the classifiable feature, and then, out of the tweets that were labeled classifiable, the rest of the classifications were trained. The weighted average *F*_1_-score of the training validation and against the blind data set was above 0.80 in all the cases except in the user and interest areas, which were slightly lower. These analyses were performed with Python 3.7 (Python Software Foundation) and using the libraries pandas, NumPy, JSON, and ktrain.

### Statistical Analysis

The frequency distribution and percentages of tweets according to different categories based on the characteristics of the tweet were reported across different tables. The comparison of the proportion of tweets between categories was carried out using the Pearson chi-square test, from which the *P* value of statistical significance is reported. Likewise, the accuracy of the different tweet distributions obtained by these multilingual machine learning models is reflected by the weighted average *F*_1_-score (a combination of precision and recall; the closer to 1, the less possibility of classification error). Retweet-to-tweet and like-to-tweet ratios according to pain drug, type of user, content, and other characteristics of the tweet were also calculated.

We used linear regression models to evaluate the associations between tweet content, type of user, and other characteristics of the tweets and the number of likes and retweets. Individual beta coefficients were adjusted for the rest of the characteristics of the tweet. These analyses were conducted with the software packages Stata (version 16; StataCorp) and Excel (Microsoft Corp).

### Ethical Considerations

This project received approval from the ethics committee of the Hospital Principe de Asturias (OE 14_2020) and is compliant with the research ethics principles of the Declaration of Helsinki (seventh revision, 2013). This study did not directly involve human participants, nor did it include any intervention; instead, it used only publicly available tweets (subject to universal access through the internet according to the terms of service that all users on Twitter accept). Nevertheless, we have taken care to not directly reveal in this report any username, and we have avoided citing tweets that could be offensive or compromised to someone.

## Results

### Patients and Health Care Professionals

Patients are the most active when it comes to talking about analgesia on Twitter; however, health care professionals are the most desired.

Of the 102,594 tweets included in the analysis, 81.03% (83,129/102,594) of the tweets were considered classifiable. The other 19,465 tweets did not include sufficient information to be classifiable (Figure 1). As shown in Table 1, the distribution of tweets was similar in both groups, with 42,840 tweets about paracetamol (42,840/83,129, 51.5% of the total) and 42,131 tweets mentioning the weak opioid group (42,131/83,129, 50.7% of the total). There were 900 tweets that mentioned on the same tweet “tramadol” and “codeine”; therefore, we included them in both groups. Another 1842 tweets mentioned paracetamol and weak opioids in the same tweet, and they are also included in both categories.

**Table 1:** Descriptive characteristics of the original tweets included in the analysis, categorized by pain drug, type of user, type of content, and other characteristics. In the pain drug category, the total sum of tweets exceeds 100% because there are 900 tweets that mentioned tramadol and codeine in the same tweet and 1842 tweets that mentioned paracetamol and tramadol or codeine in the same tweet. These tweets mentioning more than one drug were classified in both categories.

Category				Tweets, n (%)			Likes per tweet, mean (SD)			Retweet per tweet, mean (SD)
**Pain drug**										
	Paracetamol		42,840 (51.5)			4.7 (60.6)			1.0 (20.0)	
	**Weak opioids**		42,131 (50.7)			4.1 (59.5)			1.2 (25.8)	
		Tramadol	16,227 (19.5)		5.1 (63.6)			1.9 (33.9)		
		Codeine	26,804 (32.2)		3.7 (59.4)			0.8 (22.0)		
**User**										
	Patient		60,771 (73.1)			3.7 (52.4)			0.7 (19.5)	
	Family or friend		11,635 (14)			4.5 (53.4)			1.3 (23.9)	
	Health care professional		6692 (8.1)			8.6 (102.1)			2.4 (31.0)	
	Institution		4031 (4.8)			7.5 (86.4)			3.8 (37.0)	
**Content**										
	Nonmedical content		32,307 (38.9)			4.7 (63.7)			1.4 (27.5)	
	Medical content		50,822 (61.1)			4.3 (57.6)			0.9 (18.4)	
**Other characteristics**										
	**Personal opinion**		68,305 (82.2)			4.1 (59.5)			0.8 (22.1)	
		Positive	52,528 (63.2)		3.7 (53.1)			0.7 (21.5)		
		Negative	15,777 (19)		5.7 (77.0)			1.4 (24.0)		
	Query		5244 (6.3)			4.2 (59.4)			1.5 (44.1)	
Total				83,129 (100)			4.4 (60.1)			1.1 (22.4)

Regarding Twitter users, it is notable that nearly 75% (60,771/83,129) of tweets were published by users classified as patients, while health care professionals and health institutions only published 8% (6692/83,129) and 4% (4031/83,129), respectively (Table 1). However, despite having a low percentage of tweets, the tweets posted by these groups obtained the highest ratio of like-tweet and tweet-retweet. Health care professionals obtained a mean of 8.6 likes and 2.4 retweets per tweet, while tweets written by patients only obtained a mean of 3.7 likes and 0.7 retweets per published tweet (Table 1).

About the content of the tweet, nearly 2/3 (61.1%) of tweets made reference to medical content. In terms of the opinion expressed by the users, in 63% (52,528/68,305) of cases it was considered positive, 19% (15,777/68,305) negative, and the rest was undetermined (Table 1). Twitter users were asked a question in 6% (5244/83,129) of tweets.

### Efficacy of Paracetamol and Opioids

Efficacy regarding paracetamol and opioids is the most recurrent topic on Twitter; however, secondary effects generate the most interest.

Of those tweets that included medical content, 80.8% (41,080/50,822) mention the efficacy of the drug, and of those, 6.9% (3501/50,822) describe a good or sufficient efficacy. Conversely, we found that 40.7% (20,700/50,822) of tweets with medical content made mention of the adverse effects. Also, 7.2% (3661/50,822) of tweets with medical content included a reference to a scientific article. In terms of likes and retweets, the tweets that described a positive efficacy of the drug obtained a smaller number of likes and retweets per tweet than those that described a null or low efficacy (Table 2). Moreover, tweets that mentioned adverse effects generated more retweets and likes than those discussing the efficacy of the drug. Finally, the medical tweets that generated the greatest number of likes and retweets were those that made reference to scientific articles (Table 2).

Considering the nonmedical tweets, 2 out of 3 were considered to trivialize the drug. Only 1.6% (533/32,307) expressed a sentiment of solidarity or support for the users of paracetamol or weak opioids. The rest made reference to commercial or economic aspects of the drugs. Solidarity was the content that generated the highest number of retweets by far (Table 2).

**Table 2:** The number of tweets with medical and nonmedical tweets and their distribution among the different categories. In the categories of medical content, the percentages are calculated based on the total number of tweets with medical content, and in the categories of nonmedical content, the percentages are calculated based on the total number of tweets with nonmedical content.

Category					Tweets, n (%)			Likes per tweet, mean (SD)			Retweet per tweet, mean (SD)
**Medical content**											
	Fake content			8928 (18)			4.94 (56.14)			0.77 (13.93)	
	**Refers to efficacy**			41,080 (80.8)			N/A^a^			N/A	
		Little efficacy	37,579 (74)			4.14 (58.51)			0.81 (20.09)		
		Good efficacy	3501 (6.9)			2.65 (33.26)			0.48 (17.38)		
	Refers to side effects			20,700 (40.7)			5.01 (58.66)			1.27 (18.46)	
	Includes scientific link			3661 (7.2)			6.35 (76.60)			2.89 (34.02)	
	Total			50,822 (100)			4.28 (57.59)			0.85 (18.41)	
**No medical content**											
	**Commercial issues**			10,816 (33)			5.20 (61.87)			1.54 (19.24)	
		Pharmacy dispensation	3384 (10)			5.30 (61.12)			1.11 (15.16)		
		Bureaucratic difficulties	2284 (7)			5.50 (61.83)			1.03 (12.99)		
		Publicity	2358 (7)			3.63 (25.03)			1.21 (8.50)		
		Legal issues	2790 (9)			6.17 (81.56)			2.76 (30.90)		
	Economic aspects			1426 (4)			3.94 (63.75)			1.37 (27.50)	
	Solidarity			533 (1.6)			4.13 (63.75)			21.31 (140.99)	
	**Trivialization**			20,277 (63)			4.37 (63.63)			0.88 (22.35)	
		Humor	1950 (6)			6.33 (50.70)			1.02 (12.78)		
		Recreational use	7871 (24)			3.78 (43.64)			0.79 (13.14)		
		Song, poetry, or book	10,456 (32)			4.45 (77.06)			0.93 (28.43)		
	Total			32,307 (100)			4.65 (63.75)			1.37 (27.50)	

^a^N/A: not applicable.

### Patient Participation

Patients participate mostly in conversations regarding opioids but not in those talking about paracetamol.

Users classified as patients published 73.10% (60,771/83,129) of the tweets analyzed. The proportion of tweets posted by each type of user for each type of drug was significantly different. The patient group was the only one where tweets referring to opioids were more prevalent (34,609/60,771, 56.9%) than tweets referring to paracetamol (27,420/60,771, 45.1%; Table 3). For the rest of the users, the majority of tweets made reference to paracetamol, with this trend being stronger in health care professionals and health institutions, where approximately 3 of every 4 tweets made reference to paracetamol.

The distribution of medical content for each drug was statistically different (*P*<.001; Table 4). Nonmedical content was predominant (23,871/32,307, 73.9%) in tweets regarding opioids, while in paracetamol tweets, medical content was predominant (33,943/50,822, 66.8%). Statistically significant differences were also found in the distribution of medical and nonmedical content among Twitter users (Table 5). Medical content was the most predominant of all user categories. However, patients were the users with fewer medical content publications (34,144/60,771, 56.2%) and health care professionals were the subgroup with the most publications of medical content (5652/6692, 84.5%).

Within the medical content, we found statistically significant differences in the percentage of tweets that made a positive or negative reference to the efficacy as well as adverse effects between tweets concerning paracetamol and tweets concerning opioids (*P*<.001; Table 4). In the case of paracetamol, 76.1% (25,814/33,943) of tweets regarding medical content expressed a null or insufficient efficacy, while in the case of tramadol and codeine, this percentage decreased to 70.5% (7636/10,829) and 70.2% (5546/7902), respectively (Table 4). On the other hand, only 6.1% (2083/33,943) of medical tweets regarding paracetamol made reference to an adequate efficacy of the drug. Tramadol reached a higher percentage of tweets in terms of efficacy, whereas codeine reached a lower percentage (Table 4). In terms of side effects, the percentage was higher for the opioid group. The side effects of tramadol and codeine were mentioned in 44.5% (4819/10,829) and 46% (3632/7902) of medical tweets, respectively. Among the nonmedical content, we also found statistically significant differences in the distribution between the different drugs (Table 4). Commercial aspects of paracetamol and tramadol were predominant, in contrast to codeine, where it was a minority. Tweets expressing support and solidarity were the minority in the 3 drugs, while trivialization was abundant, especially in the case of opioids (Table 4). Up to 87.3% (16,493/18,902) of nonmedical tweets referring to codeine trivialized the drug.

**Table 3:** Number of tweets posted about each drug classified by type of user. The chi-square test was conducted to assess statistical differences.

Category	Total	Paracetamol	Weak opioids		
			Tramadol	Codeine	Total
Patient, n (%)	60,771	27,420 (45.1)	11,892 (19.6)	23,291 (38.3)	34,609 (56.9)
Family or friend, n (%)	11,635	7556 (64.9)	2173 (18.7)	2248 (19.3)	4280 (36.8)
Health care professional, n (%)	6692	4923 (73.6)	1343 (20.1)	717 (10.7)	1916 (28.6)
Institution, n (%)	4031	2941 (73)	819 (20.3)	548 (13.6)	1326 (32.9)
*P* value	N/A^a^	<.001	.04	<.001	<.001

^a^N/A: not applicable.

**Table 4:** Classification of tweets based on medical or nonmedical content (and its distribution among the different subcategories) and the type of drug. The chi-square test was conducted to assess statistical differences.

Category		Paracetamol	Weak opioids		
			Tramadol	Codeine	Total
**Medical content, n (%)**					
	Fake content	6546 (19.3)	725 (6.7)	1849 (23.4)	2514 (13.8)
	None or little efficacy	25,814 (76.1)	7636 (70.5)	5546 (70.2)	12,846 (70.3)
	Good efficacy	2083 (6.1)	1166 (10.8)	454 (5.7)	1584 (8.7)
	Side effects	12,809 (37.7)	4819 (44.5)	3632 (46)	8218 (45)
	Includes scientific link	2572 (7.6)	980 (9)	246 (3.1)	1153 (6.3)
	Total	33,943 (66.8)	10,829 (21.3)	7902 (15.5)	18,260 (35.9)
**Nonmedical content, n (%)**					
	Commercial issues	5971 (67.1)	3177 (58.9)	2040 (10.8)	5034 (21.1)
	Refers to a high cost	976 (11)	317 (5.9)	156 (0.8)	466 (2)
	Refers to sympathy	263 (3)	277 (5.1)	221 (1.2)	457 (1.9)
	Trivialization	2093 (23.5)	1916 (35.5)	16,493 (87.3)	18,227 (76.4)
	Total	8897 (27.5)	5398 (16.7)	18,902 (58.5)	23,871 (73.9)
*P* value		<.001	<.001	<.001	<.001

**Table 5:** Classification of tweets according to their content (medical or nonmedical) and according to the type of user who posted it. The chi-square test was conducted to assess statistical differences.

Category	Patient	Family or friend	Health care professional	Institution
Nonmedical content, n (%)	26,627 (82.4)	3316 (10.3)	1040 (3.2)	1324 (4.1)
Medical content, n (%)	34,144 (67.2)	8319 (16.4)	5652 (11.1)	2707 (5.3)
*P* value	<.001	<.001	<.001	<.001

## Discussion

### Principal Findings

In this study, we found that analgesia is a common topic of debate among Twitter users, and patients make up the majority of the participants in these discussions. Health care professionals and health care institutions, despite not being greatly represented in these discussions, tend to generate high levels of interest when they participate. Despite the severity of the matter in discussion, two-thirds of tweets with nonmedical content trivialized drugs, especially tramadol and codeine. Interestingly, in tweets related to tramadol and codeine, nonmedical content is more common, whereas in tweets related to paracetamol, medical content is more common.

Our results show that these drugs are more talked about than chemotherapy, antidiabetics, statins, antidepressants, or antipsychotics [34-36]. This finding can be explained due to the increased presence of pain. Pain can be a symptom that is present in a multitude of diseases, but it can also be a syndrome [3]. Its treatment is a topic of worry for many organizations, given that, in many cases, it is a chronic treatment. On a pharmacological level, the primary tools are opioids because paracetamol and similar drugs usually fall short in their treatment of chronic diseases [37,38]. However, opioids, including the weakest ones, are associated with gastrointestinal side effects in the short term and addiction effects in the long term [39]. The latter is a topic of concern, primarily because of the opioid epidemic that the United States is currently going through and because of the increasing use of these drugs in Western countries [40,41]. In this context, over the past few years, social media have been used as a tool for pharmaco-surveillance, and also to monitor analgesic drugs. A study that analyzed social media posts published in Pennsylvania found a correlation between the number of posts on social media suggesting opioid abuse and the data collected by the National Survey on Drug Use and Health [42]. Another study done in North Carolina found a relationship between the number of tweets and the number of deaths due to synthetic opioid and heroin overdoses [43].

Social media can be a way of accessing social groups that are less represented in traditional studies, such as young adults, Hispanic or Latin, African Americans, and women [44]. Furthermore, given that posts on social media are spontaneous, they can be a better reflection of what patients and the general public think in comparison to studies that use traditional methods [45,46]. In multiple studies, it has been shown that patients tend to show a favorable image of themselves, as well as being complacent with doctors. This results in them omitting their negative opinions and prejudices toward certain drugs to the doctor and expressing these in informal web-based forums instead, such as social media [47]. Furthermore, users on social media post what they think or experience in real time, which avoids the recall bias that is present in medical consultation [34].

Interestingly, 80% (41,080/50,822) of tweets with medical content discuss the efficacy of the drug. This is a higher percentage than what is found in other studies. For example, in a study that analyzed what Twitter users discussed regarding the efficacy of approved drugs to treat obesity, a lower percentage was found [48]. Moreover, a study with similar characteristics that analyzed what Twitter users posted regarding approved drugs for the treatment of attention-deficit/hyperactivity disorder (ADHD) also found a lower percentage of tweets discussing the efficacy of the drug in comparison to this study [49]. In another study that analyzed papers regarding psychotherapy, the percentage of tweets discussing drug efficacy was even lower [50]. These results suggest that when users on social media discuss analgesics, the main worry is the efficacy of the drug, which is not the case in other pathologies. Despite this, what is most interesting is that in this study, the majority of the tweets that reference the efficacy of the drug express that it is null or insufficient, whereas, in the case of the drugs approved for the treatment of ADHD or obesity, the majority of tweets regarding drug efficacy were positive [48,49]. On the other hand, in the study that analyzed posts regarding drugs used for the treatment of ADHD, the percentage of tweets that mentioned adverse effects was greater than the one found in this study [49]. Despite the findings in this study, the tweets that mentioned adverse effects did not receive a greater number of retweets and likes than the other posts, which is different from the findings in other areas of health. For example, a paper that analyzed posts referencing pharmacological interventions used to regulate fertility found that posts referencing adverse effects achieved the greatest retweet-tweet ratio [51]. These findings suggest that, in reference to analgesia, the public is more concerned about the efficacy of the drug. This is probably because pain is one of the most undesirable and incapacitating sensations that can be experienced [3]. However, the impression is that Twitter users underestimate the adverse effects associated with the use of opioids.

Another interesting finding in this study is the limited perception of risk perceived by users regarding the use of weak opioids. The population’s risk perception is one of the key elements when it comes to an adequate use of a drug or substance. For example, it has been estimated that the low perception of risk by adolescents in relation to cannabis use is one of the key factors explaining the high consumption rates [52,53]. This is why it is worrying that most of the tweets posted regarding tramadol and codeine had nonmedical content, which contrasts with other pharmacological groups. For example, in a study that analyzed publications on Twitter referring to antipsychotics, less than 40% of tweets mentioned nonmedical aspects of the treatment [35]. This percentage was even lower (16.32%) in publications about antidepressants [36]. The perceived risk also influences the prescription of opioids by health care professionals. In this sense, a study showed that health care professionals prescribe fewer opioids after being notified of a patient’s death due to an overdose [54].

Furthermore, we found differences in the medical content between paracetamol and weak opioids. Tramadol and codeine were considered effective in a small percentage of tweets, as well as paracetamol, which shows how complex the treatment of pain is. Drugs used to treat other diseases with a worse prognosis, such as schizophrenia, receive a better outcome in that aspect [35]. Multiple motives can explain why a high percentage of users consider these analgesics to have null or low effectiveness. In the first place, pain is a very ego-dystonic symptom. Second, it tends to appear in healthy patients, who until the start of pain, were asymptomatic and tolerated the symptom with difficulty. Third, it is very incapacitating, and as it appears in a functional patient, the impact is greater. Fourth, as there is a stronger existing treatment that is in a way more effective, the population is more demanding as it knows there is a faster alternative to relieve the pain. Finally, there are studies that have evaluated the patient´s perception of the treatment, finding that the positive outcomes of the treatment tend to be transmitted during a medical consultation, whereas the bad experiences are shared on social media or other media platforms [47].

Even though pain is an incapacitating symptom, there have been a low number of tweets demonstrating support and solidarity. This could be due to the invisibility of pain and its unknown origin in many cases. Diseases that, due to their nature or their treatment, are more visual, and we know the cause, generate greater understanding, support, and solidarity than those that are invisible or of a complex nature with social and behavioral implications [55,56]. Frequently, people tend to empathize more when a disease is more notable or manifest than when it is not. This partly explains the stigmatization of mental illness over many years. However, the low number of tweets showing compassion have been retweeted greatly. Twitter is a social media platform to launch campaigns and create awareness [57].

Finally, the theme of trivialization is a matter of concern. On multiple occasions, the trivialization of both physical and mental diseases has been described, for example, for HIV, depression, and psychosis [58-60]. Similarly, a high percentage of trivialization has been described in papers referencing antipsychotics [35]. It is necessary to create sensitizing campaigns to change this engrained mentality within the population. Perhaps this trivialization may be linked to the low percentage of professionals and institutions that have intervened in this debate. In a previous study, a low participation rate of professionals and institutions was also found in social media discussions regarding naloxone and opioids [29]. However, it is important that health care professionals and health care institutions are present in medical debates on social media, given that it is a place where many patients seek information. Particularly, in a survey carried out in the United Kingdom and Ireland, it was found that codeine users are more likely to search for help on the internet than from their doctor [61]. In fact, more than one-third of patients with chronic pain prefer to use the internet to seek information related to pain, and 60% have more confidence in the information found on the internet than the information that their doctor provides [62]. Moreover, when health care professionals intervene on Twitter, their publications are retweeted more often than other users [63]. Additionally, social media users frequently associate paracetamol with depression and suicide attempts which correlates with epidemiologic data given that its wide availability makes it a frequently used drug for self-harm [64,65].

### Limitations

This study has certain limitations. First, despite having used a search tool that has access to 100% of tweets, it is possible that tweets that referenced paracetamol, codeine, or tramadol with different keywords than those used in this study may have been left out. Second, Twitter users may include abbreviations, grammatical errors, or slang language, which makes tweet searching more difficult. Third, the succinctness of some tweets, combined with their lack of context, makes them difficult to interpret. Fourth, tweets may not be representative of the views of the rest of society. Finally, the content of tweets regarding analgesics may vary with time. Therefore, despite the time period of this study lasting 2 years, the results may not coincide with what is published today.

### Conclusions

The users most actively engaged in these conversations are the patients themselves, suggesting they resort to Twitter to express their distress and seek solutions. Patients who have pain show great interest in finding relief for their symptoms. As a result, their tweets tend to focus on the effectiveness of the drug rather than its possible side effects. This fact can pose risks, among other factors, as a minority appears content with the analgesic efficacy. From a public health point of view, it is useful that health care professionals and institutions are more involved in these discussions, being able to inform people about the effects of the analgesia. Future studies should characterize the type of pain that the users argue about in order to design more targeted interventions. Additionally, it will also be necessary to study what is being published regarding other strategies to alleviate pain, such as surgical interventions or psychotherapeutic interventions. Finally, it is important that the authorities monitor the conversations related to analgesics on social media, given that it has been demonstrated that the purchase without a prescription and the illegal sale of these drugs are common on the internet and in social media [66,67].

## Abbreviations

ADHD: attention-deficit/hyperactivity disorder
OTC: over-the-counter

## Data Availability

The data sets generated during or analyzed during this study are available from the corresponding author on reasonable request.

## References

[1] Collins FS, Koroshetz WJ, Volkow ND (2018). Helping to end addiction over the long-term: the research plan for the NIH HEAL initiative. JAMA.

[2] Coussens NP, Sittampalam GS, Jonson SG, Hall MD, Gorby HE, Tamiz AP, McManus OB, Felder CC, Rasmussen K (2019). The opioid crisis and the future of addiction and pain therapeutics. J Pharmacol Exp Ther.

[3] Cohen SP, Vase L, Hooten WM (2021). Chronic pain: an update on burden, best practices, and new advances. Lancet.

[4] Gatchel RJ, McGeary DD, McGeary CA, Lippe B (2014). Interdisciplinary chronic pain management: past, present, and future. Am Psychol.

[5] Gallagher RM (2016). Advancing the pain agenda in the veteran population. Anesthesiol Clin.

[6] (2021). Chronic Pain (Primary and Secondary) in Over 16s: Assessment of all Chronic Pain and Management of Chronic Primary Pain.

[7] Bond C, Hannaford P (2003). Issues related to monitoring the safety of over-the-counter (OTC) medicines. Drug Saf.

[8] Duong M, Gulmez SE, Salvo F, Abouelfath A, Lassalle R, Droz C, Blin P, Moore N (2016). Usage patterns of paracetamol in France. Br J Clin Pharmacol.

[9] Wolf MS, King J, Jacobson K, Di Francesco L, Bailey SC, Mullen R, McCarthy D, Serper M, Davis TC, Parker RM (2012). Risk of unintentional overdose with non-prescription acetaminophen products. J Gen Intern Med.

[10] King JP, Davis TC, Bailey SC, Jacobson KL, Hedlund LA, Di Francesco L, Parker RM, Wolf MS (2011). Developing consumer-centered, nonprescription drug labeling a study in acetaminophen. Am J Prev Med.

[11] Forman RF, Woody GE, McLellan T, Lynch KG (2006). The availability of web sites offering to sell opioid medications without prescriptions. Am J Psychiatry.

[12] Bachhuber MA, Cunningham CO (2013). Availability of buprenorphine on the internet for purchase without a prescription. Drug Alcohol Depend.

[13] Liang BA, Mackey T (2009). Searching for safety: addressing search engine, website, and provider accountability for illicit online drug sales. Am J Law Med.

[14] Nanna MG, Navar AM, Zakroysky P, Xiang Q, Goldberg AC, Robinson J, Roger VL, Virani SS, Wilson PWF, Elassal J, Lee LV, Wang TY, Peterson ED (2018). Association of patient perceptions of cardiovascular risk and beliefs on statin drugs with racial differences in statin use: insights from the patient and provider assessment of lipid management registry. JAMA Cardiol.

[15] Wei MY, Ito MK, Cohen JD, Brinton EA, Jacobson TA (2013). Predictors of statin adherence, switching, and discontinuation in the USAGE survey: understanding the use of statins in America and gaps in patient education. J Clin Lipidol.

[16] Saha K, Torous J, Kiciman E, De Choudhury M (2021). Understanding side effects of antidepressants: large-scale longitudinal study on social media data. JMIR Ment Health.

[17] Colditz JB, Chu KH, Emery SL, Larkin CR, James AE, Welling J, Primack BA (2018). Toward real-time infoveillance of Twitter health messages. Am J Public Health.

[18] Teo AR, Strange W, Bui R, Dobscha SK, Ono SS (2020). Responses to concerning posts on social media and their implications for suicide prevention training for military veterans: qualitative study. J Med Internet Res.

[19] Budenz A, Klassen A, Purtle J, Tov EY, Yudell M, Massey P (2020). Mental illness and bipolar disorder on Twitter: implications for stigma and social support. J Ment Health.

[20] Haggerty T, Sedney CL, Cowher A, Holland D, Davisson L, Dekeseredy P (2022). Twitter and communicating stigma about medications to treat obesity. Health Commun.

[21] van Schaijik B, Alshawa A, Hamadah O, Alshehri M, Kujan O (2021). The role of Twitter in dental education: a systematic review. J Dent Educ.

[22] Prutzman YM, Wiseman KP, Grady MA, Budenz A, Grenen EG, Vercammen LK, Keefe BP, Bloch MH (2021). Using digital technologies to reach tobacco users who want to quit: evidence from the National Cancer Institute's Smokefree.gov initiative. Am J Prev Med.

[23] Malik A, Khan MI, Karbasian H, Nieminen M, Ammad-Ud-Din M, Khan SA (2021). Modeling public sentiments about JUUL flavors on Twitter through machine learning. Nicotine Tob Res.

[24] Budenz A, Klassen A, Leader A, Fisher K, Yom-Tov E, Massey P (2020). HPV vaccine, Twitter, and gay, bisexual and other men who have sex with men. Health Promot Int.

[25] Massey PM, Leader A, Yom-Tov E, Budenz A, Fisher K, Klassen AC (2016). Applying multiple data collection tools to quantify human papillomavirus vaccine communication on Twitter. J Med Internet Res.

[26] Ricard BJ, Hassanpour S (2021). Deep learning for identification of alcohol-related content on social media (Reddit and Twitter): exploratory analysis of alcohol-related outcomes. J Med Internet Res.

[27] Barenholtz E, Fitzgerald ND, Hahn WE (2020). Machine-learning approaches to substance-abuse research: emerging trends and their implications. Curr Opin Psychiatry.

[28] Jashinsky J, Burton SH, Hanson CL, West J, Giraud-Carrier C, Barnes MD, Argyle T (2014). Tracking suicide risk factors through Twitter in the US. Crisis.

[29] Tofighi B, El Shahawy O, Segoshi A, Moreno KP, Badiei B, Sarker A, Krawczyk N (2021). Assessing perceptions about medications for opioid use disorder and naloxone on Twitter. J Addict Dis.

[30] Martinez B, Dailey F, Almario CV, Keller MS, Desai M, Dupuy T, Mosadeghi S, Whitman C, Lasch K, Ursos L, Spiegel BMR (2017). Patient understanding of the risks and benefits of biologic therapies in inflammatory bowel disease: insights from a large-scale analysis of social media platforms. Inflamm Bowel Dis.

[31] Golder S, Bach M, O'Connor K, Gross R, Hennessy S, Hernandez GG (2021). Public perspectives on anti-diabetic drugs: exploratory analysis of Twitter posts. JMIR Diabetes.

[32] Conneau A, Khandelwal K, Goyal N, Chaudhary V, Wenzek G, Guzmán F, Grave E, Ott M, Zettlemoyer L, Stoyanov V (2020). Unsupervised cross-lingual representation learning at scale. https://aclanthology.org/2020.acl-main.747/.

[33] Maiya AS ktrain: a low-code library for augmented machine learning. ArXiv.

[34] Golder S, O'Connor K, Hennessy S, Gross R, Gonzalez-Hernandez G (2020). Assessment of beliefs and attitudes about statins posted on Twitter: a qualitative study. JAMA Netw Open.

[35] Alvarez-Mon MA, Donat-Vargas C, Santoma-Vilaclara J, de Anta L, Goena J, Sanchez-Bayona R, Mora F, Ortega MA, Lahera G, Rodriguez-Jimenez R, Quintero J, Álvarez-Mon M (2021). Assessment of antipsychotic medications on social media: machine learning study. Front Psychiatry.

[36] de Anta L, Alvarez-Mon MA, Ortega MA, Salazar C, Donat-Vargas C, Santoma-Vilaclara J, Martin-Martinez M, Lahera G, Gutierrez-Rojas L, Rodriguez-Jimenez R, Quintero J, Alvarez-Mon M (2022). Areas of interest and social consideration of antidepressants on english tweets: a natural language processing classification study. J Pers Med.

[37] Busse JW, Wang L, Kamaleldin M, Craigie S, Riva JJ, Montoya L, Mulla SM, Lopes LC, Vogel N, Chen E, Kirmayr K, De Oliveira K, Olivieri L, Kaushal A, Chaparro LE, Oyberman I, Agarwal A, Couban R, Tsoi L, Lam T, Vandvik PO, Hsu S, Bala MM, Schandelmaier S, Scheidecker A, Ebrahim S, Ashoorion V, Rehman Y, Hong PJ, Ross S, Johnston BC, Kunz R, Sun X, Buckley N, Sessler DI, Guyatt GH (2018). Opioids for chronic noncancer pain: a systematic review and meta-analysis. JAMA.

[38] Finnerup NB, Attal N, Haroutounian S, McNicol E, Baron R, Dworkin RH, Gilron I, Haanpää M, Hansson P, Jensen TS, Kamerman PR, Lund K, Moore A, Raja SN, Rice ASC, Rowbotham M, Sena E, Siddall P, Smith BH, Wallace M (2015). Pharmacotherapy for neuropathic pain in adults: a systematic review and meta-analysis. Lancet Neurol.

[39] Xie J, Strauss VY, Martinez-Laguna D, Carbonell-Abella C, Diez-Perez A, Nogues X, Collins GS, Khalid S, Delmestri A, Turkiewicz A, Englund M, Tadrous M, Reyes C, Prieto-Alhambra D (2021). Association of tramadol vs codeine prescription dispensation with mortality and other adverse clinical outcomes. JAMA.

[40] Gostin LO, Hodge JG, Noe SA (2017). Reframing the opioid epidemic as a national emergency. JAMA.

[41] Meldrum ML (2016). The ongoing opioid prescription epidemic: historical context. Am J Public Health.

[42] Sarker A, Gonzalez-Hernandez G, Ruan Y, Perrone J (2019). Machine learning and natural language processing for geolocation-centric monitoring and characterization of opioid-related social media chatter. JAMA Netw Open.

[43] Anwar M, Khoury D, Aldridge AP, Parker SJ, Conway KP (2020). Using Twitter to surveil the opioid epidemic in North Carolina: an exploratory study. JMIR Public Health Surveill.

[44] (2018). Social media use in 2018. Pew Research Center.

[45] Lachmar EM, Wittenborn AK, Bogen KW, McCauley HL (2017). #MyDepressionLooksLike: examining public discourse about depression on Twitter. JMIR Ment Health.

[46] Berry N, Lobban F, Belousov M, Emsley R, Nenadic G, Bucci S (2017). #WhyWeTweetMH: understanding why people use Twitter to discuss mental health problems. J Med Internet Res.

[47] Leonardo N, Lester S, Graham M, Barrett C, Whittle S, Rowett D, Buchbinder R, Hill CL (2020). Selection and perception of methotrexate treatment information in people with rheumatoid arthritis. Int J Rheum Dis.

[48] Alvarez-Mon MA, Llavero-Valero M, Del Barco AA, Zaragozá C, Ortega MA, Lahera G, Quintero J, Alvarez-Mon M (2021). Areas of interest and attitudes toward antiobesity drugs: thematic and quantitative analysis using Twitter. J Med Internet Res.

[49] Alvarez-Mon MA, de Anta L, Llavero-Valero M, Lahera G, Ortega MA, Soutullo C, Quintero J, Del Barco AA, Alvarez-Mon M (2021). Areas of interest and attitudes towards the pharmacological treatment of attention deficit hyperactivity disorder: thematic and quantitative analysis using Twitter. J Clin Med.

[50] Alvarez-Mon MA, Fernandez-Lazaro CI, Ortega MA, Vidal C, Molina-Ruiz RM, Alvarez-Mon M, Martínez-González MA (2022). Analyzing psychotherapy on Twitter: an 11-year analysis of tweets from major U.S. media outlets. Front Psychiatry.

[51] Alvarez-Mon MA, Donat-Vargas C, Llavero-Valero M, Gea A, Alvarez-Mon M, Martinez-Gonzalez MA, Del Burgo CL (2021). Analysis of media outlets on women's health: thematic and quantitative analyses using Twitter. Front Public Health.

[52] Mariani AC, Williams AR (2021). Perceived risk of harm from monthly cannabis use among US adolescents: National Survey on Drug Use and Health, 2017. Prev Med Rep.

[53] Romm KF, Wang Y, Ma Y, Wysota CN, Blank MD, Huebner DM, Roche KM, Berg CJ (2022). The reciprocal relationships of social norms and risk perceptions to cigarette, e-cigarette, and cannabis use: cross-lagged panel analyses among US young adults in a longitudinal study. Drug Alcohol Depend.

[54] Doctor JN, Nguyen A, Lev R, Lucas J, Knight T, Zhao H, Menchine M (2018). Opioid prescribing decreases after learning of a patient's fatal overdose. Science.

[55] McGinty EE, Barry CL (2020). Stigma reduction to combat the addiction crisis—developing an evidence base. N Engl J Med.

[56] Volkow ND (2020). Stigma and the toll of addiction. N Engl J Med.

[57] Viguria I, Alvarez-Mon MA, Llavero-Valero M, Del Barco AA, Ortuño F, Alvarez-Mon M (2020). Eating disorder awareness campaigns: thematic and quantitative analysis using Twitter. J Med Internet Res.

[58] Alvarez-Mon MA, Llavero-Valero M, Sánchez-Bayona R, Pereira-Sanchez V, Vallejo-Valdivielso M, Monserrat J, Lahera G, Del Barco AA, Alvarez-Mon M (2019). Areas of interest and stigmatic attitudes of the general public in five relevant medical conditions: thematic and quantitative analysis using Twitter. J Med Internet Res.

[59] Robinson P, Turk D, Jilka S, Cella M (2019). Measuring attitudes towards mental health using social media: investigating stigma and trivialisation. Soc Psychiatry Psychiatr Epidemiol.

[60] Joseph AJ, Tandon N, Yang LH, Duckworth K, Torous J, Seidman LJ, Keshavan MS (2015). #Schizophrenia: use and misuse on Twitter. Schizophr Res.

[61] Kimergård A, Foley M, Davey Z, Dunne J, Drummond C, Deluca P (2017). Codeine use, dependence and help-seeking behaviour in the UK and Ireland: an online cross-sectional survey. QJM.

[62] Corcoran TB, Haigh F, Seabrook A, Schug SA (2010). A survey of patients' use of the internet for chronic pain-related information. Pain Med.

[63] Abbasi-Perez A, Alvarez-Mon MA, Donat-Vargas C, Ortega MA, Monserrat J, Perez-Gomez A, Sanz I, Alvarez-Mon M (2021). Analysis of tweets containing information related to rheumatological diseases on Twitter. Int J Environ Res Public Health.

[64] Casey D, Geulayov G, Bale E, Brand F, Clements C, Kapur N, Ness J, Patel A, Waters K, Hawton K (2020). Paracetamol self-poisoning: epidemiological study of trends and patient characteristics from the multicentre study of self-harm in England. J Affect Disord.

[65] Schück S, Roustamal A, Gedik A, Voillot P, Foulquié P, Penfornis C, Job B (2021). Assessing patient perceptions and experiences of paracetamol in France: infodemiology study using social media data mining. J Med Internet Res.

[66] Katsuki T, Mackey TK, Cuomo R (2015). Establishing a link between prescription drug abuse and illicit online pharmacies: analysis of Twitter data. J Med Internet Res.

[67] Mackey TK, Kalyanam J, Katsuki T, Lanckriet G (2017). Twitter-based detection of illegal online sale of prescription opioid. Am J Public Health.

